# Evaluation of bloodstream infections, *Clostridium difficile* infections, and gut microbiota in pediatric oncology patients

**DOI:** 10.1371/journal.pone.0191232

**Published:** 2018-01-12

**Authors:** Bryan T. Nycz, Samuel R. Dominguez, Deborah Friedman, Joanne M. Hilden, Diana Ir, Charles E. Robertson, Daniel N. Frank

**Affiliations:** 1 Division of Adult Infectious Diseases, University of Colorado School of Medicine, Aurora, Colorado, United States of America; 2 Division of Pediatric Infectious Diseases, University of Colorado School of Medicine, Aurora, Colorado, United States of America; 3 Department of Epidemiology, Children’s Hospital Colorado, Aurora, Colorado, United States of America; 4 Center for Blood and Cancer Disorders, Children’s Hospital Colorado, Aurora, Colorado, United States of America; Institut Pasteur, FRANCE

## Abstract

Bloodstream infections (BSI) and *Clostridium difficile* infections (CDI) in pediatric oncology/hematology/bone marrow transplant (BMT) populations are associated with significant morbidity and mortality. The objective of this study was to explore possible associations between altered microbiome composition and the occurrence of BSI and CDI in a cohort of pediatric oncology patients. Stool samples were collected from all patients admitted to the pediatric oncology floor from Oct.–Dec. 2012. Bacterial profiles from patient stools were determined by bacterial 16S rRNA gene profiling. Differences in overall microbiome composition were assessed by a permutation-based multivariate analysis of variance test, while differences in the relative abundances of specific taxa were assessed by Kruskal-Wallis tests. At admission, 9 of 42 patients (21%) were colonized with *C*. *difficile*, while 6 of 42 (14%) subsequently developed a CDI. Furthermore, 3 patients (7%) previously had a BSI and 6 patients (14%) subsequently developed a BSI. Differences in overall microbiome composition were significantly associated with disease type (p = 0.0086), chemotherapy treatment (p = 0.018), BSI following admission from any cause (p < 0.0001) or suspected gastrointestinal organisms (p = 0.00043). No differences in baseline microbiota were observed between individuals who did or did not subsequently develop *C*. *difficile* infection. Additionally, multiple bacterial groups varied significantly between subjects with post-admission BSI compared with no BSI. Our results suggest that differences in gut microbiota not only are associated with type of cancer and chemotherapy, but may also be predictive of subsequent bloodstream infection.

## Introduction

*Clostridium difficile* infections (CDI) are a matter of significant importance worldwide.[1–3] In both children and adults, CDI incidence is associated with health-care settings and length of stay in these facilities, as well as recent antibiotic use, immunosuppressive therapies, inflammatory bowel disease, organ transplant or malignancy, and proton pump inhibitors.[4–8] Although epidemiological studies have investigated the risk factors, incidence, and outcomes associated with CDI in adult populations, less is known in relation to pediatric populations.[9] The most frequent comorbid condition correlated with CDI in pediatric populations is cancer, with roughly 25% of cases occurring in adolescents with an underlying malignancy.[8, 10–12] Along with CDIs, bloodstream infections (BSIs) cause a significant burden of disease for pediatric oncology and bone marrow transplant (onc/BMT) patients.

Because both CDIs and BSIs result in significant morbidity, longer hospitalization, and greater financial burden among pediatric oncology patients, identifying new means to predict and prevent these infections would provide significant benefit to this at-risk population.[13, 14] Although numerous studies have investigated the incidence of CDI and BSI in children and adults, few have explored the complex relationships between *C*. *difficile*, other pathogenic organisms, and the gastrointestinal microbial community in pediatric oncology/BMT patients. A healthy microbiome, composed of diverse communities of bacteria, viruses, fungi, protozoa, and archaea, protects against pathogen colonization by competition for nutrients, niche exclusion, and ecological competition.[15] Hence, disruption of the microbiome (“dysbiosis”), resulting from cancer-induced immunodeficiency, chemotherapy treatment regimens, antibiotic use, or other factors, could increase the risk of CDI and BSI by disrupting the gut microbiome’s ability to resist pathogen colonization or by weakening the intestinal barrier, thereby promoting bacterial translocation.[16–20]

In this study, we sought to identify if a relationship exists between the human gut microbiome composition and the development of BSIs and CDIs in a small, convenience cohort of pediatric oncology patients. We hypothesized that distinct differences in the diversity and composition of gut-associated microbial communities would be observed prior to development of CDIs or BSIs following hospital admission. Furthermore, we also hypothesized that different patient types (e.g., acute myelogenous leukemia [AML], acute lymphoblastic leukemia [ALL]), along with history of chemotherapy, would manifest unique compositions of gut microbiota.

## Materials and methods

### Cohort and sample collection

The Center for Cancer and Blood Disorders (CCBD) Program, which includes inpatient and outpatient services for oncology, hematology, and bone marrow transplant patients, is located on a single floor of the free-standing Children’s Hospital of Colorado (CHCO). As previously described, as part of a *C*. *difficile* outbreak investigation, stool samples were collected from all patients admitted to the pediatric CCBD floor from October through December 2012.[9, 21] Stool samples were tested for the *C*. *difficile* toxin B gene by PCR (Xpert *C*. *difficile*, Cepheid, Sunnyvale CA). An aliquot of stool was stored at -70°C and later thawed for sequence analysis. Clinical histories were ascertained by retrospective chart review. Research and data collection protocols were approved by the Colorado Multiple Institutional Review Board. Waiver of informed consent was approved for retrospective chart review of study participants.

### Case definitions

Sample procurement, as well as inclusion and exclusion criteria are presented elsewhere.[9, 22] Previous and subsequent BSIs were defined as presence of fever with a positive blood culture within the prior month and subsequent two months of sample procurement, respectively. Previous or subsequent CDIs were defined as clinical signs of CDI (gastrointestinal symptoms and signs including diarrhea, severe abdominal pain, and/or fevers >100.4°F) and a positive stool sample for the *C*. *difficile* toxin B gene by PCR. A positive PCR result for the *C*. *difficile* toxin B gene at admission in asymptomatic patients corresponded to colonization and not CDI. Thus, CDI is defined as patients with gastrointestinal symptoms and a positive stool sample for the *C*. *difficile* toxin B gene by PCR. Colonization is defined as asymptomatic patients without gastrointestinal symptoms, but with a positive *C*. *difficile* toxin B gene by PCR. Those who were not colonized nor infected, were asymptomatic, no gastrointestinal symptoms, and with a negative *C*. *difficile* toxin B gene by PCR. Non-neoplastic, hematologic admissions (Heme) were related to acute pain crises. Three of the patients had a prior diagnosis of sickle cell anemia and the forth patient was previously diagnosed with hemophilia B and Factor X deficiency. Solid tumor types include neuroblastoma, botryoid rhabdomyosarcoma, Wilm’s tumor, retinoblastoma, osteogenic sarcoma, ovarian endodermal sinus tumor, and Burkitt’s lymphoma. Five subjects received a BMT for underlying diagnoses of recessive dystrophic epidermolysis bullosa, severe combined immunodeficiency (SCID), non-Hodgkin lymphoma, and AML. Of the five patients, only one was currently in their index BMT admission, while the remainder four patients were one and a half to ten months status post bone marrow transplant. Other reasons for admission besides fever and neutropenia, chemotherapy, and new cancer diagnosis include: acute pain events, fatigue, pallor, care follow up, and nausea, vomiting, and diarrhea.

### Microbiome analysis

Bacterial profiles were determined by broad-range analysis of 16S rRNA genes following our previously described methods.[23–26] In brief, DNA was extracted from 50–100 mg of stool using the QIAamp PowerFecal DNA isolation kit (QIAGEN Inc., Carlsbad, CA). Broad-range PCR amplicons were generated using barcoded primers that target the V3V4 variable region of the 16S rRNA gene: primers 338F (5’ ACTCCTACGGGAGGCAGCAG) and 806R (5’ GGACTACHVGGGTWTCTAAT).[27–29] PCR products were normalized using a SequalPrep^™^ kit (Invitrogen, Carlsbad, CA), pooled, and quantified by Qubit Fluorometer 2.0 (Invitrogen, Carlsbad, CA). The pool was diluted to 4nM and denatured with 0.2 N NaOH at room temperature. The denatured DNA was diluted to 15pM and spiked with 25% of the Illumina PhiX control DNA prior to loading the sequencer. Illumina paired-end sequencing was performed on the Ilumina MiSeq platform with version v2.3.0.8 of the MiSeq Control Software and version v2.3.32 of MiSeq Reporter, using a 600-cycle version 3 reagent kit.

Illumina Miseq paired-end reads were aligned to a *Homo sapiens* reference genome (UCSC Hg19) with bowtie2 and matching sequences discarded. Remaining paired-end sequences were sorted by sample via barcodes in the paired reads with a python script.[30] The sorted paired reads were assembled using phrap and pairs that did not assemble were discarded.[31] Assembled sequence ends were trimmed over a moving window of 5 nucleotides until average quality met or exceeded 20. Trimmed sequences with more than 1 ambiguity or shorter than 150 nucleotides were discarded. Potential chimeras identified with Uchime (usearch6.0.203_i86linux32) using the Schloss Silva reference sequences were removed from subsequent analyses.[32, 33] Assembled sequences were aligned and classified with SINA (1.3.0-r23838) using the bacterial sequences in Silva 115NR99 as reference configured to yield the Silva taxonomy.[34, 35] Operational taxonomic units (OTUs) were produced by clustering sequences with identical taxonomic assignments. All de-multiplexed, paired-end 16S rRNA gene sequence files, along with de-identitied clinical/demographic metadata, were deposited into the NCBI Sequence Read Archive under project number PRJNA411831.

### Statistical analysis

All data analyses were performed using the Explicet and R statistical software packages.[36, 37] The relative abundance (RA) of each taxon was calculated as the number of 16S rRNA sequences of a given taxon divided by the total number of 16S rRNA sequences in a patient’s sample. Differences in overall microbiome composition (i.e., beta-diversity) between subsets were assessed by a non-parametric permutation-based multivariate analysis of variance (PERMANOVA with 10,000 replicate resamplings) using Bray-Curtis Dissimilarities. Shannon diversity, Shannon evenness, and richness (Sobs) were calculated using rarefaction and compared across groups by analysis of variance (ANOVA) tests. Comparisons of RA across groups were conducted by Kruskal-Wallis (>2 groups) or Wilcoxon rank sum (2 groups) tests. For Kruskal-Wallis tests, if *p*-values were significant (*p* <0.05), then pairwise differences were calculated.

## Results

### Study population

In this study, we included 42 patients admitted to the pediatric oncology floor at CHCO (Table 1). Among the enrollees, 81% (n = 34) had a previous cancer diagnosis, 19% (n = 8) were first admissions for a new diagnosis of cancer, and 10% (n = 4) were hematology admissions. Furthermore, 39 of 42 subjects (92.9%) were between 2 and 22.22 years old. The 8 newly-diagnosed patients had not received any chemotherapy, whereas 11 (26%) were currently receiving chemotherapy treatment, and 23 (55%) had previously received chemotherapy. Within this cohort, 4 (10%) patients had AML, 15 (36%) had ALL, 5 (12%) had a bone marrow transplant (BMT), 14 (33%) had a solid tumor, and 4 (10%) were admitted for non-neoplastic, hematologic concerns related to acute pain events. At admission, 21% (n = 9) were colonized with *C*. *difficile*, all of whom had a cancer diagnosis preceding their current health-care encounter; 6 patients with a negative *C*. *difficile* PCR at admission subsequently developed CDI. Additionally, 3 patients (7%) had a prior BSI and 6 (14%) developed a BSI following admission. Finally, 95% (40/42) of the subjects had received antibiotics in the three months prior to stool collection.

**Table 1:** Clinical characteristics of pediatric oncology patients.

		All	*Clostridium difficile* Colonization^1^		All Bacteremia^2^	
			Positive	Negative	Positive	Negative
**Subjects**		42	21% (9)	79% (33)	21% (9)	79% (33)
**Sex (F)**		60% (25)	89% (8)	52% (17)	56% (5)	61% (20)
**Previous Cancer Diagnosis (Y)**		81% (34)	100% (9)	76% (25)	89% (8)	79% (26)
**Admission Type**						
	**New Diagnosis**	19% (8)	0% (0)	24% (8)	11% (1)	21% (7)
	**Chemotherapy**	26% (11)	33% (3)	24% (8)	44% (4)	21% (7)
	**Fever/Neutropenia**	21% (9)	7% (3)	18% (6)	11% (1)	24% (8)
	**Other**	33% (14)	7% (3)	33% (11)	33% (3)	33% (11)
**Patient Type**						
	**ALL**	36% (15)	11% (1)	42% (14)	22% (2)	39% (13)
	**AML**	10% (4)	11% (1)	9% (3)	33% (3)	3% (1)
	**BMT**	12% (5)	22% (2)	9% (3)	22% (2)	9% (3)
	**Solid**	33% (14)	44% (4)	30% (10)	11% (1)	39% (13)
	**Heme**	10% (4)	11% (1)	9% (3)	11% (1)	9% (3)
**Median 16S Reads**		84,108	109,661	80,424	84,841	83,375
**Age (years)**	**Median**	7.41	6.57	7.68	9.87	7.13
	**(Range)**	(0.31–22.2)	(0.57–20.0)	(0.31–22.2)	(2.17–22.2)	(0.31–20.3)
**BMI (kg/m2)**^3^	**Median**	17.1	18.3	17.0	17.23	17.05
	**(Range)**	(12.9–28.9)	(13.7–23.1)	(12.9–28.9)	(15.3–22.8)	(12.9–28.9)

^1^ Assessed by toxin-specific PCR of stool specimens.

^2^ Assessed by clinical blood culture

^3^ Body Mass Index (BMI)

### Community-wide alterations in microbiome composition

**Fig 1:**
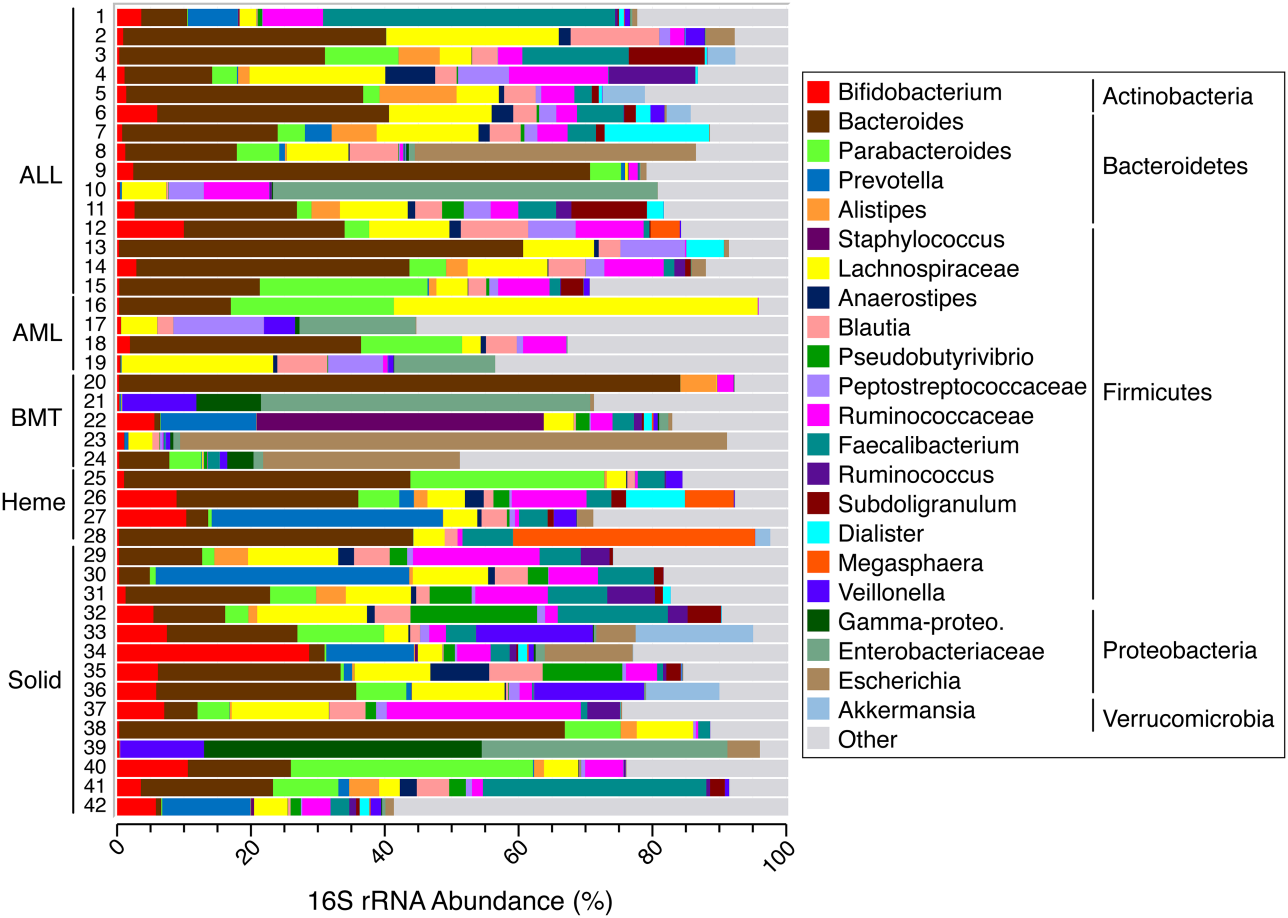
Fecal bacterial diversity among pediatric oncology cohort. Barcharts represent the percent relative abundances of genus-level taxa, as determined by 16S rRNA gene sequencing. All taxa with mean abundances across all subjects of less than 1% were aggregated into the “Other” category.

Broad-range PCR of bacterial 16S rRNA genes was successful for all 42 samples, generating a median of 84,108 (IQR 27,710–117,700) high-quality 16S sequences per sample. Regardless of clinical condition, the predominant bacterial phyla observed in this population were as expected for post-infancy fecal samples: Firmicutes (44.9% mean relative abundance [RA]), Bacteroidetes (33.7% mean RA), Proteobacteria (12.9% mean RA), and Actinobacteria (5.8% mean RA). However, substantial inter-patient variability was observed (Fig 1).

Univariable PERMANOVA tests of overall microbiome composition at the genus level (Table 2), revealed significant associations of microbiome composition with patient type (p = 0.0086), subsequent BSI (p < 0.0001), and current chemotherapy treatment (p = 0.018). When the analysis of subsequent BSI was restricted to the five cases potentially caused by gastrointestinal organisms (e.g., *Escherichia*, *Klebsiella*, *Lactobacillus*), the association with microbiome structure remained significant (p = 0.00043). Trends toward significance also were observed in *C*. *difficile* colonization at admission (p = 0.080) and admission type (p = 0.072). Both of these factors remained significant when patient type was included in the PERMANOVA analysis as a covariate (Table 2). Furthermore, except for the *C*. *difficile* colonization results, similar results were attained when the data were analyzed at the phylum level (Table 2), indicating broad-level changes in microbiome composition across patient groups.

**Table 2:** Associations between clinical/demographic variables and gastrointestinal microbiome^1^.

	P-Value (Genus-level)		P-Value (Phylum-level)	
Patient Variable^2^	Unadjusted	Adjusted for Patient Type	Unadjusted	Adjusted for Patient Type
**Patient Type**	0.0086	na	0.044	na
**Admit Type**	0.072	0.052	0.038	0.035
**New Dx**	0.40	0.34	0.30	0.28
**Hem Admit**	0.53	0.45	0.41	0.36
**Chemotherapy**	0.018	0.012	0.0049	0.0038
**Sex**	0.98	0.97	0.92	0.90
**Neutropenia**	0.15	0.13	0.30	0.26
**Severe Neutropenia**	0.44	0.38	0.55	0.51
***C*. *difficile***:				
Previous CDI	0.67	0.48	0.90	0.86
Admit (Colonization)	0.080	0.048	0.69	0.62
Subsequent CDI	0.24	0.16	0.33	0.30
**BSI**:				
Ever	0.0026	0.0019	0.00013	<0.0001
Previous	0.73	0.73	0.71	0.74
Subsequent	<0.0001	<0.0001	<0.0001	<0.0001
Subsequent GI	0.00043	0.00026	0.00024	0.00016

^1^ P-values are the results of PERMANOVA tests at the indicated taxonomic rank.

^2^ Definitions of patient groups are provided in the text.

Because microbiota composition was significantly associated with patient type, we next performed a PERMANOVA analysis of each pairwise combination of patient types (Table 3). In the genus-level analysis, the microbiota of ALL patients differed significantly from AML (p = 0.040) and BMT (p = 0.0061) patients, and trended towards significance compared with patients with solid tumors (p = 0.084). AML and BMT patients also differed significantly from solid tumor patients (p = 0.051 and p = 0.037, respectively). Phylum-level comparisons also were significant for a subset of these comparisons (Table 3).

**Table 3:** Differences in gastrointestinal microbiome between patient types^1^.

	**ALL**	**AML**	**BMT**	**Heme**	**Solid**
**ALL**	---	0.040	0.006	0.24	0.084
**AML**	0.024	---	0.23	0.14	0.051
**BMT**	0.030	0.40	---	0.26	0.037
**Heme**	0.28	0.29	0.19	---	0.65
**Solid**	0.52	0.090	0.069	0.65	---

^1^ P-values are the results of univariable PERMANOVA tests.

The top-right triangle presents genus-level results, whereas the bottom-left triangle presents phylum-level results.

### Alterations in microbial diversity

**Fig 2:**
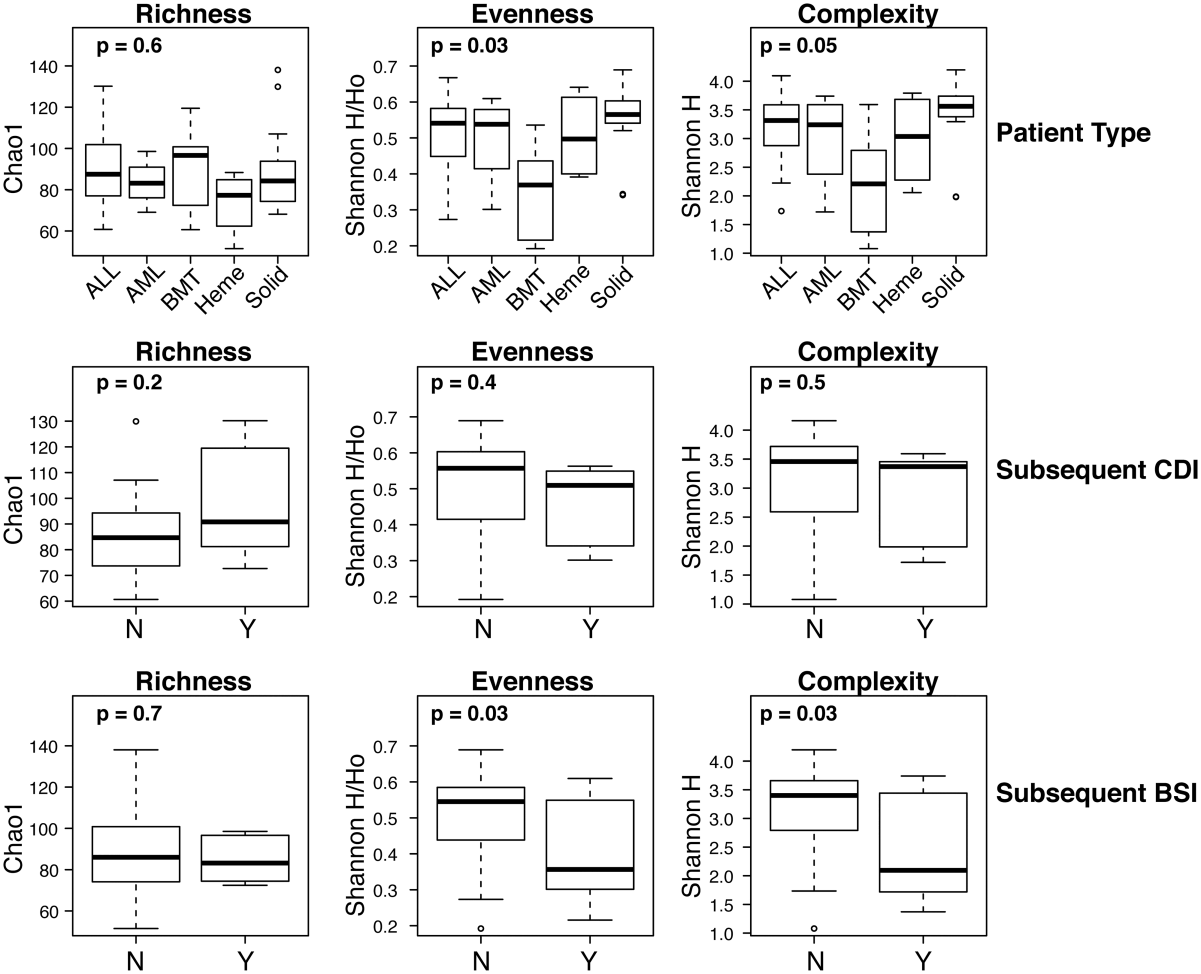
Alpha biodiversity indices in association with patient characteristics. Richness, evenness, and complexity (i.e., Shannon Diversity) were inferred from 16S rRNA sequence datasets through rarefaction and replicate resampling. P-values across all groups were ascertained by ANOVA. Abbreviations for patient types are detailed in the text. “Subsequent *C*. *diff*” compared subjects who had no history of *C*. *difficile* infection with those who recorded an infection following admission. Similarly, “Subsequent BSI” compared subjects who had no history of bloodstream infection with those who recorded an infection following admission. ALL: acute lymphoblastic leukemia; AML: acute myelogenous leukemia; BMT: bone marrow transplant; Heme: hematology; Solid: neuroblastoma, rhabdomyosarcoma, Wilm’s tumor, retinoblastoma, osteogenic sarcoma, ovarian endodermal sinus tumor, etc.

Gut microbiota differed significantly across patient types in the alpha-diversity measures of community evenness (the uniformity of OTU distributions estimated by Shannon H/H_max_; p = 0.034) and complexity (Shannon diversity; p = 0.053), while richness (the estimated number of OTUs per sample) was unchanged (Fig 2).[38, 39] These effects were due primarily to BMT patients, who manifested lower evenness and complexity compared with other patient types. No differences in alpha diversity were noted between individuals who either developed *C*. *difficile* infection or remained free of *C*. *difficile* (Fig 2, middle panels). In contrast, individuals who developed BSI following fecal sample collection harbored significantly less diverse gut microbiotas, as measured by both evenness (p = 0.030) and complexity indices (p = 0.027), compared with those who were never diagnosed with BSI (Fig 2, bottom panels). These findings remained significant following adjustment for patient type (data not shown). No differences in alpha diversity were noted in relation to chemotherapy status, admission type, or new diagnosis (data not shown).

### Bacterial groups differing by patient characteristics

**Fig 3:**
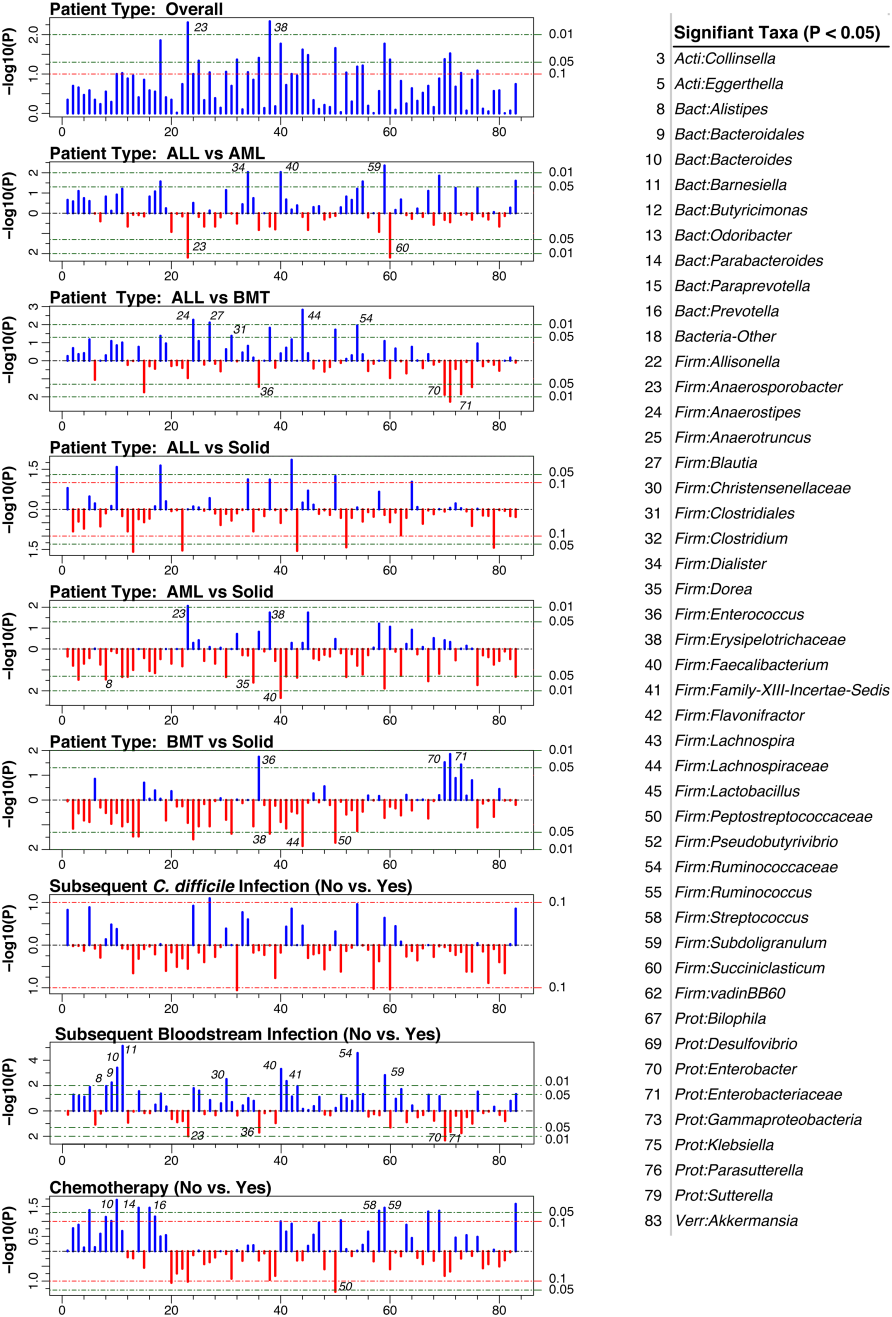
Significant taxa. Manhattan plots display the p-values for each OTU along the x-axis, following Kruskal-Wallis tests of relative abundance. P-values are plotted on the y-axis following–log10 transformation. OTUs that are significant in one or more statistical test are listed to the right of the plots. Horizontal lines denote p-values of 0.1, 0.05 or 0.01. Taxa that are either discuss in the text or that had p < 0.01 are labeled on each plot.

To identify individual bacterial groups that differed in abundance in association with patient variables, we applied the non-parametric Kruskal-Wallis test to the genus-level microbiota data, focusing on the significant variables identified through PERMANOVA analysis (i.e., patient type, *C*. *diff* colonization/infection, BSI, chemotherapy). Results are presented in Fig 3 as Manhattan plots, which plot a transformed p-value (-log10(p-value)) for each taxon tested (because of the exploratory nature of this study, p-values were not corrected for multiple comparisons). Multiple genera were identified as significant in one or more comparison.

Subjects with AML vs. ALL differed in several bacterial taxa, including *Dialister* (p = 0.0080), *Faecalibacterium* (p = 0.0093), and *Subdoligranulum* (p = 0.0044), each of which was decreased in abundance in AML compared with ALL. In constrast, *Anaerosporobacter* (p = 0.0065) and *Succiniclasticum* (p = 0.0065) were increased in AML. As for AML vs Solid, significant changes were observed in *Faecalibacterium* (p = 0.0046; decreased in AML), *Alistipes* (p = 0.034; decreased in AML), *Dorea* (p = 0.026; decreased in AML), *Anaerosporobacter* (p = 0.0087), and *Erysipelotrichaceae* (p = 0.019; increased in AML). For ALL vs BMT, significant changes were observed in *Lachnospiraceae* (p = 0.0015; increased in ALL), *Ruminococcaceae* (p = 0.013; increased in ALL), *Clostridiales* (p = 0.042; increased in ALL), *Enterobacter* (p = 0.013; increased in BMT), *Enterobacteriaceae* (p = 0.0054; increased in BMT), and *Enterococcus* (p = 0.036; increased in BMT). For BMT vs Solid, significant alterations were observed in *Lachnospiraceae* (p = 0.016; increased in Solid), *Peptostreptococcaceae* (p = 0.021; increased in Solid), *Erysipelotrichaceae* (p = 0.042; increased in Solid), *Enterobacter* (p = 0.030; increased in BMT), *Enterobacteriaceae* (p = 0.014; increased in BMT), and *Enterococcus* (p = 0.018; increased in BMT). No taxa were found to differ between patients with *C*. *difficile* infection following admission and those who were never colonized or infected.

Noteworthy changes in patients with bloodstream infections following admission, compared with those who did not develop an infection, were observed in *Bacteroides* (p = 0.00039; decreased in BSI), *Ruminococcaceae* (p < 0.00010; decreased in BSI), *Alistipes* (p = 0.011; decreased in BSI), *Faecalibacterium* (p = 0.00049; decreased in BSI), *Enterobacter* (p = 0.022; increased in BSI), *Enterobacteriaceae* (p = 0.022; increased in BSI), and *Enterococcus* (p = 0.020; increased in BSI) (Table 2 and Fig 3). Of note, individuals who developed BSI had low *Bacteroides* (0.05% relative abundance) and high *Enterobacteriaceae* (16.2%) compared to those who did not develop BSI (21.6% and 0.02%, respectively). No statistical difference between groups was observed for viridans group streptococci, which frequently cause BSIs among pediatric oncology populations.

Significant alterations in patients who were currently receiving chemotherapy treatment, compared with those who were not receiving chemotherapy were observed in several bacterial taxa, including *Peptostreptococcaceae* (p = 0.044), *Streptococcus* (p = 0.044), *Parabacteroides* (p = 0.035), *Prevotella* (p = 0.034), and *Subdoligranulum* (p = 0.034) (Table 2 and Fig 3). Patients receiving chemotherapy treatment showed decreased microbial abundance within each of these taxa, except for *Peptostreptococcaceae*, which showed increased abundances.

### Detection of bloodstream pathogens in fecal samples

We hypothesized that increased burdens of selected fecal organisms might precede their occurrence in the bloodstreams of patients who experienced BSIs following admission. A total of 11 bacterial species were identified in blood cultures from the 6 subjects who developed a BSI following admission; the parental genera of 7 of these species were detected in fecal specimens by 16S rRNA gene sequencing while the remaining 4 were not detected (Table 4). In 3 of these subjects, at least one of the genera (*Klebsiella* for case BN04, *Escherichia* for BN36, and *Streptococcus* for BN43) had a fecal abundance that was notably greater than the 75% quartile for that genus across all subjects. BSIs occurred an average of 34 days following stool collection, with the earliesy at 2 days and the latest at 82 days.

**Table 4:** Fecal 16S rRNA gene abundance of pathogens cultured from bloodstream infections occurring following admission.

			Fecal 16S rRNA Rel. Abundance (%)	
Subject	Days elapsed from stool collection to BSI	BSI Culture Results	Case^1^	All Subjects^2^
**BN01**	61	*Staphylococcus haemolyticus*	0.0021	<1e-04 (0.000–0.005)
	80	*Viridans streptococcus*	0.37	0.26 (0.066–0.70)
**BN04**	21	*Staphylococcus epidermidis*	Not detected^3^	<1e-04 (0.000–0.005)
	65	*Klebsiella pneumoniae*	**3.13**	8e-04 (0.000–0.022)
**BN16**	2	*Staphylococcus haemolyticus*	Not detected^3^	<1e-04 (0.000–0.021)
**BN25**	8	*Stenotrophomonas maltophilia*	**0.00057**	<1e-04 (0.000–<1e-04)
**BN36**	12	*Gemella morbillorm*	Not detected^3^	0.001 (0.000–0.002)
	82^4^	*Escherichia coli ESBL*	**4.85**	0.056 (0.006–0.862)
	82^4^	*Staphylococcus aureus*	0.0011	<1e-04 (0.000–0.005)
**BN43**	5	*Viridans Streptococcus Group*	**7.37**	0.26 (0.066–0.70)
	35	*Klebsiella pneumoniae*	Not detected	8e-04 (0.000–0.022)

^1^ Relative 16S rRNA gene abundance of genus that included the pathogen species cultured from the specified subject. Values greater than the 75% quartile are shown in bold.

^2^ Median (Interquartile range) relative abundance for all subjects.

^3^ Zero 16S rRNA gene sequences assigned to the genus.

^4^Positive BSI result from same clinical blood draw procedure.

## Discussion

Among the pediatric oncology patients included in this study, we identified significant associations between fecal microbiome composition and development of subsequent bloodstream infections, patient type, and chemotherapy treatment. Similar to the results of Rajagopala et al., individuals receiving chemotherapy treatment at the time of sample collection exhibited decreased abundances of several bacterial genera and/or phyla.[40] It is unclear whether microbiome changes in oncology patients are due to the cancer itself, neoplastic specific treatments, or other potential factors, such as particular antibiotic regimes (95% of the patients received some type of antibiotic in the three months prior to stool specimen collection). Regardless of the underlying cause, these alterations were associated with deleterious outcomes. Our study also observed significant changes in microbial diversity between ALL vs AML, ALL vs BMT, and BMT vs Solid tumor patients, while it did not uncover a significant difference between the microbiotas of AML and ALL patients from those of hematology and/or solid tumor patients, as assessed by PERMANOVA tests. However, we did identify several individual taxa that differed in abundance between these groups (AML and ALL versus solid tumors and hematology admissions). Furthermore, patients who developed a subsequent BSI were characterized by significantly reduced biodiversity indices (i.e., complexity and evenness) compared with those who did not develop an infection.

Approximately 5% of pediatric cancer patients develop CDI during their treatment and multiple hospital visits.[8, 10–12] Tai et al. reported that the incidence rate of CDI among hospitalized pediatric oncology patients was over 15 times greater than that of individuals without cancer.[12] Another multicenter, retrospective cohort study determined that 11% of newly diagnosed pediatric patients with acute myelogenous leukemia (AML) had CDI, and in these children, there was a 14% recurrence rate.[41] We previously reported that approximately one-third of pediatric oncology patients tested upon hospital admission were colonized with *C*. *difficile*, as indicated by a positive PCR test in the absence of gastrointestinal symptoms. We also found that more than half of oncology patients with a history of CDI who were tested during a follow-up period of up to 20 weeks after diagnosis remained intermittently or persistently colonized with *C*. *difficile* following antibiotic treatment. Interestingly, several of the patients had different *C*. *difficile* strains over time, suggesting acquisition of new strains or carriage of multiple strains simultaneously. Furthermore, none of the newly diagnosed oncology patients were colonized with *C*. *difficile* at admission, suggesting a high acquisition rate of *C*. *difficile* from healthcare encounters.[9] However, we were unable to identify any changes in baseline microbiota that were predictive of subsequent *C*. *difficile* infection.

Similar to CDIs, bloodstream infections (BSIs) are a common and potentially life threating problem for pediatric oncology and bone marrow transplant (onc/BMT) patients.[42] A multicenter study conducted by Gaur et al. reported that the majority of CLABSIs (64%) in pediatric oncology patients occurred in those with leukemia, and of these individuals, 60% had AML.[43] Additionally, Rogers et al. reported that nearly two-thirds of all AML patients developed at least one CLABSI during therapy. In contrast to the era before central line care bundles, when most CLABSIs resulted from central line complications, Rogers et al. found that the majority of pathogens causing CLABSIs in AML patients were from microorganisms commonly found in the oral cavity or gastrointestinal tract.[44]

Our data suggests that changes in the gastrointestinal microbiota may predispose and contribute to the development of bacteremia in pediatric oncology and BMT patients. Translocation could be due to either decreased mucosal integrity due to damage associated with chemotherapy or other medical interventions (radiation, immunosuppressive agents, etc.) or a compromised immune system that is unable to prevent pathogen entry into the lymphatic and blood circulatory systems. We recently reported that most pathogens causing CLABSIs in pediatric patients with AML were not skin bacteria traditionally associated with line infections, but microorganisms commonly found in the oral cavity and gastrointestinal tract.[45] Specifically, 78.2% of CLABSIs resulted from *Enterobacter*, *Streptococcus*, *Klebsiella* or *Escherichia spp*. Interestingly, individuals who developed BSI following admission harbored significantly different fecal microbiota (PERMANOVA p < 0.0001), characterized by increased abundances and prevalences of *Gammaproteobacteria*, *Enterobacteriaceae*, and *Enterococcus* at the time of admission, compared with patients who did not develop BSIs (Fig 3). Furthermore, the data provide some limited support for our hypothesis that blooms of particular species might be responsible for their increased translocation across the intestinal epithelial barriers and into the bloodstream (Table 4). Because BSIs occurred an average of 34 days following stool collection, it is possible that fecal sampling nearer in time to BSIs might have detected such blooms. Furthermore, fecal sampling would not necessarily detect expanded populations of bacterial groups in more proximal regions of the alimentary canal.

In addition to its potential mediating role in infectious diseases, the human microbiome has increasingly been linked to carcinogenesis.[46–51] Although the exact pathways are not well-established, multiple studies have proposed unique mechanisms contributing to pathology including microbial byproducts inducing and promoting cancer states, as well as microbial-induced inflammatory states.[52–55] Similarly, dysbiosis is likely induced by cancer therapies. For example, Montassier et al. demonstrated that chemotherapy treatment in an adult population significantly altered the abundance and prevalence of gut microbes.[56] This group also reported significant dysregulation of host homeostasis coinciding with microbial dysbiosis in relation to various biochemical mechanisms and products important to host health.[56] Although little is known in relations to pediatric populations, Rajagopala et al. demonstrated that pediatric oncology patients with acute lymphoblastic leukemia (ALL) undergoing multiple rounds of therapy exhibited decreased microbial diversity compared with a control population.[40] Additionally, Wang et al. elicited that the oral microbiome of patients with ALL, compared with healthy counterparts, demonstrate reduced diversity and abundance.[57]

In relation to each patient type and its corresponding microbiota, we did not identify patterns of altered taxa that were shared across different patient types, apart from two taxa with increased abundance (*Anaerosporobacter* and *Erysipelotrichaceae*). Rather, each patient type exhibited distinct differences in relation to the other patient groups. These disease-specific and/or treatment-specific microbiotas may represent unique biomarkers that potentially could be used for early cancer detection or treatment monitoring. Further studies would need to be conducted to confirm this hypothesis. As for *Erysipelotrichaceae*, multiple studies have demonstrated that this family is associated with various inflammatory and metabolic disorders, as well as key metabolic processing pathways and neoplastic conditions.[58–61] Of importance to our study, Dinh et al. reported that *Erysipelotrichaceae* was associated with increased levels of tumor necrosis factor alpha (TNF-α), an important pro-inflammatory and apoptotic mediator in chronic HIV infection.[58, 62] Therefore, *Erysipelotrichaceae* may similarly contribute to local or systemic inflammation in oncology patients, negatively influencing intestinal barrier structures, and thereby contributing to a leaky gut, bacterial translocation, and a resultant BSI.

Several limitations of this study must be noted. First, this study involved only a pilot cohort and convenience samples collected for a previously conducted outbreak investigation.[9, 21] Second, because of the cross-sectional study design, we cannot make any definitive claims of causation for any factors (i.e., chemotherapy, patient type, antibiotic usage, etc.) associated with dysbiosis. Furthermore, differences based upon amount and type of chemotherapy received cannot be separated from patient type. Other limitations include using patients with solid tumors or hematologic conditions as controls, lack of healthy controls, and lack of accessibility to patient medical records from outside CHCO’s network (treatments, medications, therapies, recent illnesses, etc.). Due to the relatively small cohort size, we did not analyze the effects of specific chemotherapeutic or antibiotic treatment regimens, both of which may have confounded results. Finally, longitudinal, prospective studies are necessary to provide additional support for the reported associations.

In summary, our results suggest that differences in fecal microbiome composition may be predictive of subsequent bloodstream infections in pediatric oncology patients. Prospective studies are needed to further explore these relationships. If future studies support these associations, clinicians might be able to develop novel strategies to predict, prevent, and better treat these infections by following changes in the intestinal microbiome and/or utilizing preventative strategies or targeted therapies against intestinal dysbiosis.
