# Correction: Evaluation of bloodstream infections, *Clostridium difficile* infections, and gut microbiota in pediatric oncology patients

**DOI:** 10.1371/journal.pone.0197530

**Published:** 2018-05-10

**Authors:** Bryan T. Nycz, Samuel R. Dominguez, Deborah Friedman, Joanne M. Hilden, Diana Ir, Charles E. Robertson, Daniel N. Frank

There is an error in Table 1. Please see the correct Table 1 here.

**Table 1:** Clinical characteristics of pediatric oncology patients.

		All	*Clostridium difficile* Colonization^1^		All Bacteremia^2^	
					Positive	Negative	Positive	Negative
**Subjects**		42	21% (9)	79% (33)	21% (9)	79% (33)
**Sex (F)**		60% (25)	89% (8)	52% (17)	56% (5)	61% (20)
**Previous Cancer Diagnosis (Y)**		81% (34)	100% (9)	76% (25)	89% (8)	79% (26)
**Admission Type**						
**New Diagnosis**		19% (8)	0% (0)	24% (8)	11% (1)	21% (7)
**Chemotherapy**		26% (11)	33% (3)	24% (8)	44% (4)	21% (7)
**Fever/Neutropenia**		21% (9)	33% (3)	18% (6)	11% (1)	24% (8)
** **	**Other**	33% (14)	33% (3)	33% (11)	33% (3)	33% (11)
**Patient Type**						
**ALL**		36% (15)	11% (1)	42% (14)	22% (2)	39% (13)
**AML**		10% (4)	11% (1)	9% (3)	33% (3)	3% (1)
**BMT**		12% (5)	22% (2)	9% (3)	22% (2)	9% (3)
**Solid**		33% (14)	44% (4)	30% (10)	11% (1)	39% (13)
**Heme**		10% (4)	11% (1)	9% (3)	11% (1)	9% (3)
**Median 16S Reads**		84,108	109,661	80,424	84,841	83,375
**Age (years)**	**Median (Range)**	7.41 (0.31–22.2)	6.57 (0.57–20.0)	7.68 (0.31–22.2)	9.87 (2.17–22.2)	7.13 (0.31–20.3)
**BMI (kg/m2)**^**3**^	**Median (Range)**	17.1 (12.9–28.9)	18.3 (13.7–23.1)	17.0 (12.9–28.9)	17.23 (15.3–22.8)	17.05 (12.9–28.9)

1 Assessed by toxin-specific PCR of stool specimens.

2 Assessed by clinical blood culture

3 Body Mass Index (BMI)
